# Molecular Characterization of XX Maleness

**DOI:** 10.3390/ijms20236089

**Published:** 2019-12-03

**Authors:** Romina P. Grinspon, Rodolfo A. Rey

**Affiliations:** 1 Centro de Investigaciones Endocrinológicas “Dr. César Bergadá” (CEDIE), CONICET – FEI – División de Endocrinología, Hospital de Niños Ricardo Gutiérrez, C1425EFD Buenos Aires, Argentina; 2 Departamento de Histología, Biología Celular, Embriología y Genética, Facultad de Medicina, Universidad de Buenos Aires, C1121ABG Buenos Aires, Argentina

**Keywords:** testis, ovary, disorders of sex development

## Abstract

Androgens and anti-Müllerian hormone (AMH), secreted by the foetal testis, are responsible for the development of male reproductive organs and the regression of female anlagen. Virilization of the reproductive tract in association with the absence of Müllerian derivatives in the XX foetus implies the existence of testicular tissue, which can occur in the presence or absence of SRY. Recent advancement in the knowledge of the opposing gene cascades driving to the differentiation of the gonadal ridge into testes or ovaries during early foetal development has provided insight into the molecular explanation of XX maleness.

## 1. Introduction

**Figure 1:**
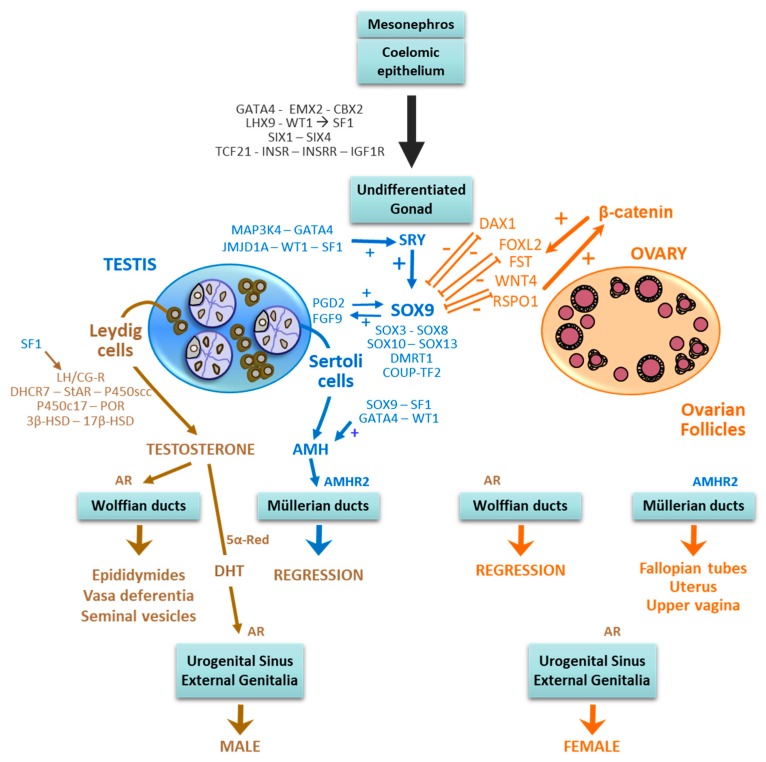
Sex differentiation during embryonic and foetal life in mammals: the mesonephros and the coelomic epithelium are stabilized and their cells proliferate, in response to the action of GATA4, EMX2, CBX2, LHX9 and WT1 -which upregulate SF1-, SIX1 and SIX4, TCF21 and members of the IGF family, to form the undifferentiated gonad. In XY foetuses, SRY expression is triggered by MAP3K4, GATA4, JMJDA1, WT1 and SF1; SRY upregulates SOX9 and other SOX family members. SOX9, PGD2 and FGF9 establish a positive feed-forward loop, which increases SOX9 expression, which prevails over DAX1, FOXL2, WNT4 and RSPO1, thus promoting testicular differentiation. The developing testis secretes testosterone and anti-Müllerian hormone (AMH), responsible for male differentiation of internal and external genitalia; testosterone can be transformed into dihydrotestosterone (DHT), a more potent androgen, through the action of the enzyme 5α-reductase (5α-Red); both testosterone and DHT act on the same androgen receptor (AR). In the XX foetuses, due to the absence of SRY, SOX9 expression remains low and is overcome by DAX1, FOXL2, WNT4 and RSPO1, which upregulate β-catenin; a feed-forward loop between these pro-ovarian factors is established, resulting in the differentiation of the female gonad. Since the ovary does not secrete androgens and AMH, internal and external genitalia develop through the female pathway. Modified with permission from Rey and Grinspon, 2011 [5]. Copyright 2010 Elsevier Ltd.

Ovarian differentiation and female internal and external genitalia are the expected pathway in the mammalian XX foetus (Figure 1). Only rarely, may XX gonads follow the testicular differentiation pathway, and consequently internal and external genitalia are virilised by testicular hormones. This “sex-reversal” condition was initially characterised in humans and named “XX male” [1], since no hint of a disorder of sex development was present until adulthood, when these males sought medical attention for infertility (see below). Alternatively, testicular tissue was also observed together with ovarian tissue in the same individual carrying an XX karyotype. The condition was known previously as “hermaphroditism”, and most frequently internal and external genitalia are ambiguous since the testicular moiety is functionally insufficient to induce full virilisation during embryonic and foetal development. Finally, virilisation of an XX foetus with ovarian development and absence of testicular tissue can be the consequence of an androgen excess of extragonadal origin. Indeed, disorders of adrenal steroidogenesis—like congenital adrenal hyperplasia [2], androgen-secreting adrenal or ovarian tumours or maternal use of anabolic steroids [3], and placental aromatase deficiency [4] can lead to foetal virilisation in the absence of testicular tissue. In this review, we will address only those conditions in which there is testicular differentiation despite an XX karyotype, with special emphasis in conditions affecting human individuals.

## 2. Molecular Mechanisms Underlying Foetal Sex Differentiation

### 2.1. The Sexually Undifferentiated Stage

Irrespective of their sex-chromosome constitution, all mammalian embryos are anatomically, histologically and functionally undifferentiated from a sexual standpoint during the first stages of development, approximately six weeks in humans and 10 days in mice. The gonadal ridges are bipotential, i.e., they can form ovaries or testes. Concomitantly, XX and XY embryos develop the anlagen for both male and female internal reproductive ducts; each pair of ducts is unipotential: the Wolffian (mesonephric) ducts may give rise only to male functional reproductive derivatives, whereas, the Müllerian (paramesonephric) ducts may give rise only to female functional structures. The primordia of the external genitalia are bipotential, like the gonads.

During the bipotential period, the XX and the XY gonadal ridges show identical expression profiles for purportedly pro-testicular factors, like SOX9 and FGF9, and pro-ovarian factors, like WNT4, RSPO1, and DAX1 [6]. Cell proliferation in the somatic component of the gonadal ridges is an essential process, which is regulated at this stage by LHX9 interaction with WT1 resulting in the modulating SF1 expression [7,8]. Other factors responsible for somatic cell proliferation are TCF21, INSR, IGF1R, and INSRR (Figure 1). During early gonadal differentiation, cell proliferation is more critical in the male testis than in the ovary [9]. WT1 and SF1 play also key roles in the whole process of urogenital organogenesis. WT1, named after Wilms’ tumour (nephroblastoma), is a transcriptional and post-transcriptional regulator [10] that is expressed early in the urogenital ridge and plays an essential role in the development of the kidneys and gonads. SF1 is encoded by the *NR5A1* gene. Initially described as a regulator of steroid hydroxylases in the adrenal cortex, SF1 is an orphan nuclear receptor also expressed in the hypothalamus, the pituitary and the gonads [11]. In the early embryo, SF1 is essential for the development of the gonadal primordium [12].

### 2.2. Testicular Differentiation

The differentiation of the gonadal ridges into ovaries or testes requires a delicate dosage balance in the timing and levels of expression of several genes [13]. In most mammalian embryos, the transient expression of *SRY*, which maps to the Y chromosome, triggers a cascade of gene interactions ultimately leading to the formation of a testis from the indifferent gonadal ridge [14]. SRY expression initiates in the middle of the gonad and expands toward the poles [15]. The timing and level of SRY expression are critical for proper testis differentiation: delayed or decreased expression results in dysgenetic testicular or ovotesticular differentiation in the mouse [16,17,18]. In most mammals, the SRY-box gene *SOX9* is the earliest upregulated gene in the testis pathway downstream of *SRY* (Figure 1), followed by *CITED4* and other members of the SOX family, including *SOX3*, *SOX8, SOX10* and *SOX13*. Many other genes are also critical for testicular differentiation [13,19,20]. *FGF9* has a role in Sertoli cell differentiation: *Fgf9*-knockout mice have dysgenetic gonads [21].

In human foetuses, Sertoli cell differentiation, characterised by the expression of SOX9 [22], AMH [23,24] and DHH [25,26,27], and cord formation begin in the central part of the gonad [28] by the end of the seventh week. Differentiating Sertoli cells also express growth factors, like nerve growth factors (NGFs), which can induce cell migration from the mesonephros acting through their receptors TRKA (NTRK1) and TRKC (NTRK3) [29,30]. In the interstitial compartment, Leydig cells differentiate amongst connective tissue and blood vessels. The formation of the coelomic vessel is a particular feature of early testicular differentiation [31,32].

The origin of Leydig cells is less clear: the mesonephros, the coelomic epithelium, the neural crest and resident SF1-expressing cells in the adreno-gonadal primordium have been proposed as their precursors [33,34,35,36,37]. Leydig cells differentiate a few days later than Sertoli cells initially depending, at least partially, on Sertoli cell-secreted PDGFs binding to PDGFRα [38] independently of gonadotropin action [39,40]. Nonetheless, in humans further Leydig cell differentiation requires placental chorionic gonadotropin (hCG) in the first and second trimesters of foetal life and foetal pituitary luteinizing hormone (LH) thereafter acting on the LH/CG receptor [41]. FGF9 [42] and DHH [43] are other factors secreted by Sertoli cells and responsible for normal Leydig cell differentiation.

### 2.3. Ovarian Differentiation

Genes involved in ovarian differentiation of the bipotential gonad increase their expression somewhat later than those involved in testis differentiation. WNT4 and RSPO1 stabilise β-catenin, encoded by *CTNNB1*, which promotes the expression of ovarian genes, like *FST* (follistatin) and *FOXL2* (Figure 1), resulting in granulosa cell differentiation [44,45,46]. Follistatin also counteracts FGF9, SOX9 and activins A and B, thus repressing the formation of the coelomic vessel [13,47,48,49,50]. By upregulating DAX1, WNT4 also antagonizes SF1 and thus inhibits androgen biosynthesis [51]. WNT4 also acts as a germ cell survival factor in the ovary [52]. In the mouse, FOXL2 is required to continuously suppress SOX9 until adulthood, thus preventing ovarian cells from transdifferentiating into “testis-like” cells [53]. Finally, a number of factors are essential for folliculogenesis [54,55]: neurotrophins and their receptors promote follicular development, FIGα is essential for primordial follicle formation, NOBOX, SOHLH1 and SOHLH2 play a role in the differentiation to primary follicles, and BMP15 and GDF9 are involved in later stages of folliculogenesis.

### 2.4. The Differentiation of the Internal and External Genitalia

The ground-breaking observations made by Jost almost seventy years ago [56] taught us that the differentiation of the gonadal ridges into testes is determinant for the virilisation of the foetus. This is due to the fact that the testes have two endocrine cell populations: Leydig cells produce androgens, which induce the differentiation of the Wolffian duct into the epididymis, vas deferens and seminal vesicles and the virilisation of the primordia of the external genitalia [57], and Sertoli cells secrete anti-Müllerian hormone (AMH), responsible for the regression of the Müllerian ducts, which otherwise form the Fallopian tubes, the uterus and the upper vagina [58,59] (Figure 1).

AMH acts very early in foetal life through its interaction with the specific AMH type 2 receptor, encoded by *AMHR2* [60]. The molecular mechanisms involved in this process have recently been reviewed in detail [61]. On the other hand, testosterone and dihydrotestosterone [62], acting through the androgen receptor, drive the differentiation of derivatives of the Wolffian duct, the urogenital sinus and the external genitalia. The extent of the regression of Müllerian ducts as well as of the male differentiation of the internal and external genitalia is commensurate with the functional capacity of the foetal testes to produce AMH and androgens during the narrow window of foetal sex differentiation [5].

## 3. Pathogenesis of XX Maleness

**Figure 2:**
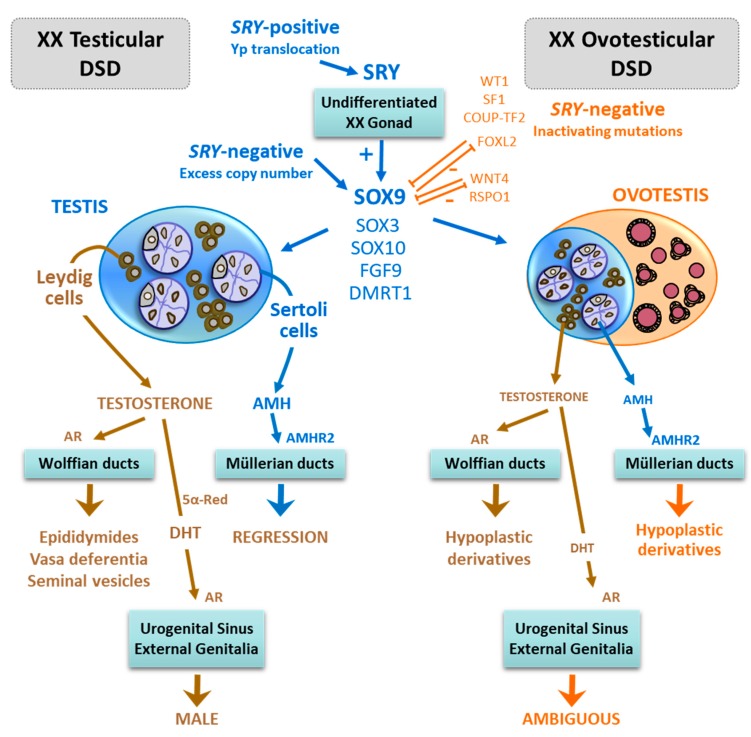
Testicular and ovotesticular differentiation in XX foetuses. In individuals with Yp translocations, the presence of SRY triggers testicular differentiation (*SRY*-positive cases). In *SRY*-negative individuals, testicular development may result from overexpression of “pro-testicular” factors (e.g., SOX9, SOX3, SOX10, FGF9, DMRT1) due to duplications/triplications of gene coding or regulatory sequences, or from inactivating mutations of “pro-ovarian” factors (e.g., RSPO1, WNT4, FOXL2) or factors that are believed to favour the gene dosage balance through the ovarian pathway (WT1, SF1, COUP-TF2). Generally, though not always, testicular DSD presents with male genitalia and ovotesticular DSD with ambiguous genitalia.

As it can be deduced from the knowledge of the normal mechanisms underlying foetal sex differentiation described above, testicular tissue can occur in individuals when Y-chromosome sequences encompassing *SRY* are present (Figure 2). These individuals, known as *SRY*-positive, may have a pure 46,XX karyotype or 46,XX/46,XY chimaerism. Nonetheless, testicular development may take place in the absence of SRY, these cases being described as *SRY*-negative. Finally, male development of the external genitalia can occur in the absence of testicular tissue, for instance in human patients with congenital adrenal hyperplasia, androgen-secreting adrenal or ovarian tumours, maternal use of anabolic steroids or placental aromatase deficiency. These are beyond the scope of the present review.

In both *SRY*-positive and *SRY*-negative situations, the gonads can completely differentiate into testes, and the condition is known as “XX male” or “46,XX testicular disorder of sex development (DSD)” (Figure 2). When testicular and ovarian tissues are present, as confirmed by histological studies showing the presence of seminiferous tubules and ovarian follicles with oocytes, the condition is known as “hermaphroditism” (term used widely in previous decades but now limited to animals, see below) or “ovotesticular DSD” (preferred for humans) (Figure 2). The proportions of ovarian and testicular tissue can vary amongst patients, and the presentation may also be asymmetric, with predominance of one tissue type on one side and of the other contralaterally, or even the existence of only one tissue type on one side and only the other or both contralaterally. As discussed below, the amount of functional testicular tissue determines the anatomical aspect of the internal and external genitalia, while the amount of ovarian tissue has no effect on the genital phenotype of the new-born.

## 4. Clinical Aspects

### 4.1. Males

In humans, and according to the nomenclature adopted after by the Chicago consensus [63], XX maleness refers to 46,XX testicular DSD. The original concept of “XX male” was used to describe patients with. “a male phenotype, male psychosexual identification, testes or gonads of testicular type without macroscopic or microscopic evidence of ovarian tissue, and absence of female genital organs” [64]. Approximately, 90% of XX males are *SRY*-positive [65]. If testes have differentiated normally during foetal development, clinical features of XX males are expected to be similar, irrespective of the presence or absence of SRY.

Typical cases usually go underdiagnosed at birth and during infancy. More rarely, these boys may seek medical attention for short stature or for small genitalia at the age of puberty. Indeed, 46,XX boys are shorter than age-matched XY pairs, with a stature in the range of age-matched girls. The underlying pathogenic mechanisms seems to be related to the existence of growth genes on the Y chromosome, rather than on growth hormone axis alterations [66]. Gonadal size is normal during childhood, because in this period testicular volume essentially depends on Sertoli cell number [67], which is not affected in XX males. Sertoli and Leydig cell function is normal during childhood and the early stages of puberty in 46,XX males, as revealed by normal testis hormone and gonadotropin levels [68,69]. Conversely, testes remain smaller than normal during puberty and adulthood, as in patients with Klinefelter syndrome, [65]. Testicular dysfunction becomes evident after pubertal onset, when the normal increase in testicular volume is led by germ cell proliferation. The existence of two X chromosomes results in a spermatogenic failure due to dramatic germ cell loss during meiosis. Therefore, these patients are azoospermic [70]. Sertoli cell markers, like inhibin B and AMH are extremely low or undetectable, and FSH is elevated. Leydig cell function may be preserved, although a mild dysfunction can exist leading to a mild hypoandrogenism with increased LH. As in patients with Klinefelter syndrome, oestrogen levels can be high and responsible for the development of gynaecomastia [65,71].

### 4.2. Ambiguous Genitalia

Virilisation during foetal life, with varying degrees of genital ambiguity, is the consequence when the mass or functional capacity of the testicular tissue is reduced—as compared to that of the normal testis—in patients with testicular or ovotesticular DSD (Figure 2). As already mentioned, the differential diagnosis between ovotesticular and testicular DSD is histologic. The clinical presentation is similar to that of other types of DSD and may range from an almost completely male presentation with mild hypospadias and cryptorchidism to an almost fully female genital phenotype with clitoromegaly and fusion of the labia minora. As discussed above, the degree of virilisation of the internal genitalia correlates with that of the external genitalia; Müllerian derivatives are present in less virilised patients and can be asymmetrical, if the amount of functional testicular tissue is larger on one side.

The circulating levels of AMH and androgens are usually below the male range but high for females [68,69]. Oestradiol levels may be indicative of the existence of functional ovarian tissue in new-borns and in patients of pubertal age. Gonadotropins are usually within the normal range, probably reflecting the action of ovarian oestrogens in the latter case, since testicular tissue is dysgenetic in most cases [68,72]. FSH elevation is indicative of scarce gonadal tissue.

While the general considerations for sex assignment in DSD apply in these cases [63,73], a particular issue derives from the fact that the ovarian tissue may yield oocyte output after pubertal development in patients with ovotesticular DSD, which raises the possibility of fertility. Thus, assignment to the female gender may be preferred in these cases, and preservation of ovarian tissue should be encouraged during surgical procedures. Testicular tissue needs to be removed to avoid virilisation during puberty. Less than twenty cases of pregnancy have been reported in patients with ovotesticular DSD; as a consequence of reproductive tract abnormalities, preterm labour and morbidity related to the delivery process are frequent [74].

In patients with ovotesticular DSD raised as boys, the ovarian tissue needs to be removed before pubertal age to prevent potential complications of cystic follicles resulting from exposure to elevated FSH, and to avoid exaggerated gynaecomastia due to oestrogen exposure. Also, in rare cases, patients raised as males and not fully diagnosed in childhood, have been reported to present with cyclical haematuria as teenagers. Subsequent workup with ultrasonography, cystourethrogram and/or cystoscopy revealed the existence of a uterus and a vagina draining into the penile urethra [75].

The risk of tumour development in the testicular tissue seems to be low, even if there are clear signs of gonadal dysgenesis. This is most likely due to the absence of peri-centromeric sequences of the Y chromosome [76].

## 5. Genetic aspects

In blood samples of patients with ovotesticular or testicular DSD, 46,XX is the most frequently found karyotype, ranging between 65% and 90% [77,78,79]. The remaining cases carry a Y chromosome (46,XX/46,XY, 46,XY, or mosaicisms with a Y-cell lineage), explaining the development of testicular tissue. The absence of a Y chromosome in the peripheral blood karyotype may be due to an imbalanced distribution of cell lines in individuals resulting in hidden mosaicisms. Although the existence of pericentromeric Y chromosomal sequences is usually associated with an increased risk for gonadal germ cell tumours in patients with DSD and gonadal signs of dysgenesis, the precise tumour risk in patients with 46,XX/46,XY chimaerism or mosaicisms carrying Y chromosome is not known.

### 5.1. SRY-Positive

*SRY*, which encodes a transcription factor belonging to the high mobility group (HMG)-box family of DNA binding proteins, maps to Yp11.2, adjacent to the pseudoautosomal region 1 (PAR1) of the Y chromosome [80]. This makes it susceptible to abnormal translocation to the short arm of the X chromosome, where the homologous PAR1 region lies. Thus, the *SRY* gene may be present in the X chromosome in 46,XX individuals with testicular or ovotesticular DSD. Less frequently, SRY may be abnormally translocated to an autosome. The actual prevalence of *SRY*-positive forms of 46,XX DSD is difficult to appraise, owing to inclusion and/or ascertainment biases in the literature. While some authors have reported a frequency of 10% of *SRY*-positive cases in 46,XX testicular or ovotesticular DSD [81], others have found up to 100% [77]. Nonetheless, it is clear that *SRY*-positive forms present more frequently (>90%) as individuals with 46,XX testicular DSD and complete virilisation, i.e., the originally described “XX males” [65,82], and far less frequently (< 10%) as patients with 46,XX ovotesticular or testicular DSD and ambiguous genitalia [82]. Differences in the reported existence of *SRY* sequences between blood and gonadal tissue may exist as a consequence of hidden mosaicisms [83].

### 5.2. SRY-Negative with Increased Expression of Pro-Testicular Genes

The differentiation of testicular tissue in an *SRY*-negative XX gonad may be due to two main mechanisms, the increased expression of pro-testicular genes or the insufficient expression of pro-ovarian/anti-testicular genes (Figure 2). In some recently described gene mutations, the pathogenic mechanism involved an imbalance of genes that participate in early gonadal development.

#### 5.2.1. SOX9

SOX9 is a transcription factor belonging to the SOX (SRY-related HMG box) family of transcriptional regulators containing a highly conserved high mobility group (HMG) domain, initially identified in SRY [84]. *SOX9* maps to 17q24.3 in the human and depicts multi-tissue expression, including Sertoli cells in the testes. SOX9 may act as a transcriptional activator or repressor [85]. In the mouse, SOX9 expression is induced in the gonadal ridge by SRY, which establishes a positive feedback loop by inducing the expression of FGF9 and PGD2 (Figure 1). This mechanism explains why SOX9 expression continues in the testis after SRY expression declines in late foetal life in the mouse. SOX9 is crucial for Sertoli cell differentiation [86], where it initiates AMH expression [87,88]. The need for a double dose of SOX9 expression for normal testicular development is illustrated by the occurrence of gonadal dysgenesis in 46,XY individuals with SOX9 haploinsufficiency [85,89,90].

On the other hand, increased expression of SOX9 in XX gonads induces testicular differentiation resulting in foetal virilisation even in the absence of *SRY* (Table 1). Experimental evidence confirming the implication of excess or ectopic SOX9 expression arises from transgenic XX mice with testis development and male genitalia [91]. The characterisation of the adult gonads in these transgenic XX mice with excess *Sox9* dosage showed the existence of seminiferous tubules without spermatogenesis, as expected in a male with two X chromosomes.

Interestingly, duplications or triplications of *SOX9* regulatory sequences, potentially explaining SOX9 overexpression, have also been described in *SRY*-negative XX individuals with virilisation [94,95,96,97,99,101,103,104,105]. Experimental evidence indicates that the regulatory region of *SOX9* is spread over at least a 2 Mb upstream of the coding sequence [85,100,106], where multiple tissue-specific enhancers exist [107]. Recently, conserved SOX9 testis-specific enhancers have been characterised (eALDI, eSR-A and eSR-B): they are deleted in 46,XY patients with dysgenetic DSD and duplicated in 46,XX *SRY*-negative patients with ovotesticular or testicular DSD [85,100]. The minimal overlap duplication is a 24 kb region [104]. Approximately, half of these patients were normally virilised, with small testes and infertility due to azoospermia in adulthood, while the others presented with ambiguous genitalia. When histology was available, at least one of the gonads was an ovotestes.

#### 5.2.2. SOX3

SOX3, another member of the SRY-related proteins, is encoded by a single exon gene mapping to Xq27.1. SOX3 is not essential for male gonadal development [108], but it provokes testicular differentiation when overexpressed in the embryonic XX gonads [109]. The underlying mechanism is the synergistic action between SOX3 and SF1 on SOX9 expression, thus activating Sertoli cell differentiation in the XX gonadal ridge (Table 2).

Concordantly, duplications of the *SOX3* locus result in testicular tissue development leading to partial or complete virilisation of the genitalia in 46,XX *SRY*-negative individuals [96,109,110,111]. However, the existence of a *SOX3* gene duplication may not suffice to provoke testicular differentiation: this could be explained by selective inactivation of the X chromosome carrying the duplication or the lack of the regulatory sequences that enhance *SOX3* expression in the duplicated region [119].

As for *SOX9*, testicular or ovotesticular DSD has been described in patients with rearrangements of *SOX3* regulatory regions. In four patients, there was a duplication of 206 kb corresponding to a region more than 500 kb upstream of *SOX3* transcription start site [112], and in another case, a 774-kb autosomal (chromosome 1) translocation into a palindromic sequence 82 kb distal to *SOX3* was associated with ovotesticular DSD characterised by the presence of a testis on one side and an ovary contralaterally [113].

#### 5.2.3. SOX10

*SOX10* maps to chromosome 22q13.1 in humans and is expressed with a similar profile to SOX9. Like SOX3, SOX10 has not been clearly demonstrated to have a functional role in normal testicular differentiation [120]. It is suspected that SOX3 and SOX10 act redundantly in embryonic testis development, which may explain why loss-of-function of one of them does not generate an abnormal phenotype [121]. Nevertheless, SOX10 overexpression in the gonadal ridges of transgenic XX mice drives the gonads through the male pathway [121]. In humans, duplications of chromosome 22 encompassing the *SOX10* locus have been found in *SRY*-negative 46,XX cases with testicular or ovotesticular DSD [114,115,116,117,121] (Table 2). External genitalia were male or ambiguous, and multiple congenital defects were found, like intrauterine growth retardation, cleft palate or heart anomalies. A recent publication reported a 46,XX male patient with a 22q11.2q13 duplication who also had the four components of PCWH: Peripheral demyelinating neuropathy, Central demyelinating leukodystrophy, Waardenburg syndrome and Hirschsprung Disease [117].

#### 5.2.4. FGF9

*FGF9* gene is located on chromosome 13q11-q12. Factors of the FGF family are involved in the development of various organs, including limbs, lungs, the adenohypophysis and the gonadal ridges. *Fgf9* knockout XY mice have seriously impaired gonadal development during embryonic and foetal life, resulting in reproductive phenotypes ranging from hypovirilisation to completely female [21]. A study of 46,XX male patients with testicular azoospermia identified one 44,XX *SRY*-negative case presenting with hypospadias in whom a duplication of 13q12.11, encompassing the entire *FGF9* gene, was detected (Table 2). The duplication was supposed to result in FGF9 overexpression, which could explain testicular rather than ovarian development [118].

#### 5.2.5. DMRT1

*DMRT* genes, mapping to chromosome 9p24.3 in humans, are characterised by the DM domain, a conserved DNA-binding motif [122]. *DMRT1* encodes a male-specific transcription factor involved in testicular differentiation in vertebrates, through the activation of many testicular genes and the downregulation of ovarian genes [123]. Deletions of chromosome 9p that include the *DMRT* cluster have been described in 46,XY DSD patients with dysgenetic testes [124] and overexpression of DMRT1 in the gonadal ridges has been shown to induce testicular differentiation and male sex development in transgenic XX mice [123,125], but *SRY*-negative 46,XX DSD attributable to *DMRT1* upregulation has not been described in humans.

### 5.3. SRY-Negative with Insufficient Expression of Pro-Ovarian Genes

#### 5.3.1. WNT4

*WNT4* maps to chromosome 1p36.12 and has many roles in foetal development of reproductive organs: it participates in ovarian differentiation and in Müllerian duct formation [48]. WNT4 is a secreted glycoprotein that reduces β-catenin phosphorylation and degradation, by cooperating with RSPO1. The increase in β-catenin levels antagonises SOX9, via the upregulation of DAX1, which antagonises SF1 [48,50]. XX mice knockout for *Wnt4* are virilised, with Wolffian duct development and absence of Müllerian ducts [126].

In humans, WNT4 overexpression was described in a 46,XY DSD patient with female phenotype, although the gonadal tissue could not be studied [127], and heterozygous loss-of-function mutations were reported in three 46,XX patients with mild virilisation. [128,129], although no testicular tissue could be identified [130]. Conversely, loss-of-function of both *WNT4* alleles results in SERKAL syndrome, a lethal condition, characterised by *SRY*-negative 46,XX testicular or ovotesticular DSD (Table 3), adrenal hypoplasia, renal agenesis and severe defects of lungs and cardiovascular structures [131].

#### 5.3.2. RSPO1

*RSPO1*, mapping to human chromosome 1p34.3, encodes a protein of the R-spondin family, widely expressed during foetal development [132]. RSPO1 synergises with WNT4 in XX gonads to stabilize β-catenin, which is essential for normal ovarian development [48]. In XX mice, the gonadal phenotype of the *Rspo1* and the *Wnt4* knockouts are strikingly similar [132]: it ranges from small testes to ovotestes [133]. It is interesting to note that the masculinisation of the foetal gonad can be rescued by β-catenin overexpression [133].

In humans (Table 3), patients from five families have been reported to date with *RSPO1* mutations associated with *SRY*-negative 46,XX DSD, palmoplantar hyperkeratosis and squamous cell carcinoma of skin [134,135,136,137,138,139]. Congenital microphthalmia, cataracts, coloboma of iris and choroid, onychodystrophy and hearing impairment were also present in some of the cases. All of them had ambiguous genitalia; in most of the patients, gonadal histology was not reported, but the presence of ovotestis was described in one patient [136] and of dysgenetic testes in another one [138].

#### 5.3.3. FOXL2

*FOXL2*, a single-exon gene mapping to 3q22.3 in humans, encodes a forkhead/winged-helix transcription factor. In most vertebrates, FOXL2 is one of the earliest markers of foetal ovarian differentiation, and is required for female sex determination in goats [140]. In mice, although FOXL2 does not seem essential for ovarian development, overexpression in the XY foetus impairs testis differentiation [141]. FOXL2 expression persists during postnatal life, and its ablation in the adult provokes transdifferentiation of the ovaries to testes [53]. In humans, virilising forms of DSD have not been described; instead, 46,XX patients with *FOXL2* loss-of-function present with the blepharophimosis-ptosis-epicanthus inversus syndrome (BPES) with or without ovarian dysgenesis but no development of testicular tissue [142].

### 5.4. SRY-Negative with Mixed or Unknown Pathogenic Mechanisms

#### 5.4.1. NR5A1 (SF1)

*NR5A1* maps to 9q33.3 in humans and encodes SF1, also called Ad4BP and FTZF1, a nuclear receptor with key roles in gonadal development and function. In the XY embryo, SF1 acts synergistically with SRY to initiate the male developmental pathway through upregulation of SOX9. In the XX embryo, in the absence of SRY, SF1 induces WNT4 and RSPO1 expression, leading to upregulation of FOXL2 and other ovary-specific factors as well as anti-testis factors, like DAX1.

Loss-of-function mutations in *NR5A1* are associated with a wide spectrum of conditions in 46,XY individuals, including complete or partial testicular dysgenesis with ambiguous genitalia, female genitalia or male infertility, and with primary ovarian insufficiency in 46,XX women [143].

In the last few years, a novel and recurrent heterozygous missense variant, c.274C > T (p.Arg92Trp) in exon 4 of *NR5A1* was identified in *SRY*-negative 46,XX testicular or ovotesticular DSD patients [143,144,145,146,147,148,149,150] (Table 4).

The hypothesized mechanism is that this novel mutation affects the activation of female-specific factors, such as WNT4/β-catenin, leading to decreased FOXL2 expression, which can no longer prosecute its pro-ovarian functions. At the same time, male-promoting genes escape firm suppression, ultimately resulting in *NR5A1*-mediated and/or independent SOX9 upregulation which triggers testicular differentiation [144,151]. Intriguingly, the same variant was found in 46,XX unaffected individuals suggesting incomplete penetrance. Recently, a 46,XX patient has been reported with ovotesticular DSD due to a paternally inherited, heterozygous variant in the same position of *NR5A1* but involving a different amino-acid, p.Arg92Gln [152].

This broad range of phenotypes emphasizes that correct SF1 function is essential for both male and female gonadal development and maintenance.

#### 5.4.2. WT1

*WT1*, which maps to human chromosome 11p13, encodes a DNA-binding protein containing four zinc fingers, which is essential for normal mammalian urogenital development. Classically, its pathogenic variants are associated with anomalies of testis and renal development, leading to 46,XY DSD and early childhood cortico-resistant nephrotic proteinuria followed by renal failure, and Wilms’ tumour [153]. The risk of or gonadoblastoma is increased in patients with WT1 defects as compared to all other cases of DSD.

An *SRY*-negative 46,XX patient with testicular DSD case has been recently reported harbouring a novel and de novo frameshift WT1 p.R485G variant [154]. The patient presented with ambiguous genitalia, characterised by clitoromegaly, a single perineal opening and a short blind-ending vagina, and dysgenetic testes. The underlying pathogenic mechanism is believed to be explained by an imbalance between the female and male gonadal determination pathways, based on a study showing that the WT1 is required for the lineage specification of Sertoli cells of the testis and granulosa cells of the ovary. Lack of WT1 in the gonadal ridge would lead to overexpression of SF1 resulting in a reduction of FOXL2 (pre-granulosa) cells and an increase in SOX9 (pre-Sertoli) cells [155].

#### 5.4.3. NR2F2 (COUP-TF2)

*NR2F2*, mapping to 15q26.2 in humans, encodes the transcription factor chicken ovalbumin upstream promoter transcription factor 2 (COUP-TF2), which acts as a human ‘‘pro-ovary’’ and ‘‘anti-testis’’ factor in the gonadal ridges and has many other morphogenetic functions, especially in the cardiovascular system [156].

A recent study described three patients with a syndromic form of testicular and ovotesticular 46,XX DSD that includes genital virilization, congenital heart disease and somatic anomalies such as BPES and congenital diaphragmatic hernia, associated with NR2F2 mutations. The three patients presented frameshift mutations affecting the N-terminal region of the protein [157]. The mechanism responsible for testis development associated with COUP-TF2 variants remains to be defined. COUP-TF2 may be required to establish ovary identity during early human gonad development by the repression of genes involved in testis determination. A major role in the mesenchymal-epithelial transition explains the congenital heart anomalies.

## 6. Concluding Remarks and Unresolved Questions

Male differentiation can occur in XX individuals in the presence or the absence of *SRY*. The advent of genomic technologies has represented an important driving force for the discovery of new mechanisms explaining the development of testicular tissue in the absence of SRY expression in the undifferentiated gonads during foetal life. While the overexpression of “pro-testicular” genes, like SOX family members, can be easily understood to explain that testicular determination depends on several genes, the lack of expression of “anti-testicular” and “pro-ovarian” genes requires the use of more complex strategies to produce evidence in order to unequivocally explain the underlying mechanism. Furthermore, mutations in genes hitherto considered as neither “pro-testis” nor “pro-ovary”, like *WT1* and *NR5A1*, have expanded the spectrum and triggered the exploration of unsuspected pathways.

## Figures and Tables

**Table 1:** Increased expression of SOX9 causing *SRY*-negative testicular or ovotesticular differentiation in XX individuals.

Genetics	Ref.	Genitalia	Gonads	Molecular Findings
Duplication of *SOX9* gene	[92]	Ambiguous	Scrotal Histol. NA	~8-cM duplication dup(17)(q23.1q24.3) involving the entire *SOX9* gene
	[93]	Male	Scrotal Histol. NA	Duplication extends at least 5 kb upstream and downstream of *SOX9*
Duplication/Triplication of *SOX9* regulatory sequences	[94]	Male	Scrotal Testes	178-kb duplication 600 kb upstream of *SOX9*
	[95]	Male	Scrotal Testes	96-kb triplication 514 kb upstream of *SOX9*
	[96]	Pt 1: Male Pt 2: Ambiguous Pt 3: Male	Pt 1: Histol. NA Pt 2: Scrotal. Bilateral ovotestes Pt 3: Histol. NA	Pt 1: 364-kb duplication 294–658 kb upstream of *SOX9* Pt 2: 141-kb duplication 31–572 kb upstream of *SOX9* Pt 3: Reciprocal translocation t(11;17)(p13;q24.3).
	[97]	Ambiguous	Ovotestes/Testes	Pt 1: 143-kb duplication 516–659 kb upstream of *SOX9* Pt 2: 444-kb duplication 259–703 kb upstream of *SOX9* Pt 3: At least 480-kb duplication 264–744 kb upstream of *SOX9*
	[98]	Ambiguous	One scrotal testis Histol. NA	Translocation t(12;17)(q14.3;q24.3) involving regulatory elements of pseudogene *LOC204010* or of gene *Deynar* 776–811 kb upstream of *SOX9*
	[99]	Ambiguous	Pt 1. Scrotal, Histol. NA Pt 2: Scrotal testis and abdominal ovary Pt 3: Scrotal ovotestis and abdominal streak	Pt 1: 605–694-kb duplication 353 kb upstream of *SOX9* Pt 2: 148-kb duplication 447–595 kb upstream of *SOX9* Pt3. 762–780-kb duplication 508 kb upstream of *SOX9*
	[100]	NA	Pt 1: Bilateral testes Pt 2: Ovotestes	Duplicated minimum critical region of 5.2 kb located ~600 kb upstream of *SOX9*, common to Pt 1 and Pt 2
	[101]	Male	Pt 1 and Pt 2: Testicular dysgenesis Pt 3: Histol. NA	Duplicated minimum critical region of ~41 kb located ~600 kb upstream of SOX9, common to all 3 patients
	[102]	Ambiguous	Ovary and ovotestis	Duplication from −581 kb upstream of to 4.4 kb coding sequence of *SOX9*
	[103,104]	Male	Left Scrotal Ovotestis Right ectopic gonad (Histol. NA)	Duplicated minimum critical region of 24 kb located ~600 kb upstream of *SOX9*
	[105]	Hypospadias	Scrotal Histol. NA	74-kb duplication 510–584 kb upstream of SOX9

Pt: Patient Histol. NA: Histology not available.

**Table 2:** Increased expression of SOX3, SOX10 and FGF9 causing *SRY*-negative testicular or ovotesticular differentiation in XX individuals.

Genetics	Ref.	Genitalia	Gonads	Molecular Findings
Duplication of *SOX3* gene	[109]	Pt 1 and Pt 2: males Pt 3: scrotal, retractile testes	Histol. NA	Pt 1: Microduplication of ~123 kb involving the entire *SOX3* gene Pt 3: ~6-Mb duplication involving the entire *SOX3* gene
	[96]	Male	Histol. NA	5.6-Mb duplication involving the entire *SOX3* gene
	[110]	Ambiguous	Abdominal ovotestes	~500-kb duplication involving the entire *SOX3* gene
	[111]	Ambiguous	Scrotal Histol. NA	494-kb duplication involving the entire *SOX3* gene
Rearrangement of *SOX3* regulatory regions	[96,109]	Male	Scrotal Dysgenetic testes	343-kb microdeletion immediately upstream of *SOX3*
	[112]	Ambiguous	Testes	Duplicated minimum critical region of ~41 kb located ~566 kb upstream of SOX3, common to all 4 patients
	[113]	Ambiguous	Scrotal One testis, one ovary	~774-kb insertion of chromosome 1 ~80 kb downstream of *SOX3* gene
*SOX10* duplication	[114]	Ambiguous	One testis, one ovary	Duplication involving the entire *SOX10* gene
	[115]	Male, micropenis	Cryptorchid testes	Trisomy of chromosome 22, where *SOX10* maps
	[116]	Male	Scrotal Histol. NA	Partial duplication of chromosome 22q including the entire *SOX10* gene
	[117]	Male	Scrotal Histol. NA	22q11.2q13 duplication, including the entire *SOX10* gene
*FGF9* duplication	[118]	Ambiguous	Histol. NA	Duplication of 13q12.11, where *FGF9* maps

Pt: Patient Histol. NA: Histology not available.

**Table 3:** Decreased expression of WNT4 and RSPO1 causing *SRY*-negative testicular or ovotesticular differentiation in XX individuals.

Genetics	Ref.	Genitalia	Gonads	Molecular Findings
*WNT4* mutations	[131]	Pt 1: Ambiguous Pt 2: Male + SERKAL syndrome	Pt 1: Dysgenetic testes Pt 2: Ovotestes	*WNT4* gene: c.C341T (p.A114V)
*RSPO1* mutations	[134,135]	Ambiguous	Histol. NA	Family 1: *RSPO1*: c.108_109insG (frameshift mutation in exon 5) Family 2: Homozygous deletion of 2752 bp (exon 4 and adjacent introns)
	[136]	Ambiguous	Ovotestes + Seminoma	*RSPO1:* homozygous splice-donor-site mutation c.28611G > A
	[137]	Ambiguous	Histol. NA	*RSPO1:* c.43_43delA (frameshift mutation in exon 4)
	[138]	Ambiguous	Pt 1: Dysgenetic testes Pt 2: Not reported	*RSPO1:* homozygous mutation c.332G > A (p.Cys111Tyr)

Pt: Patient Histol. NA: Histology not available.

**Table 4:** Mutations of NR5A1, encoding SF1, WT1 and NR2F2, encoding COUP-TF2, causing *SRY*-negative testicular or ovotesticular differentiation by unexplained mechanisms.

Genetics	Ref.	Genitalia	Gonads	Molecular Findings
*NR5A1 mutations*	[140]	Ambiguous (*n*:3)	Pt 1: Dysgenetic testis and fibrous streak Pt 2: Bilateral ovotestes Pt 3: Bilateral dysgenetic testes	*NR5A1*: c.274C > T (p.Arg92Trp)
	[141]	Family 1 (*n*:.2): Ambiguous. Family 2 (*n*:1): Micropenis, bilateral cryptorchidism Family 3 (*n*:1): Male	Family 1: Ovotestes. Family 2: Histol. NA Family 3 (*n*:1) Testes	*NR5A1*: c.274C > T (p.Arg92Trp)
	[139]	Ambiguous	Scrotal Histol. NA	*NR5A1*: c.274C > T (p.Arg92Trp)
	[143,146]	Ambiguous	Pt 1: Dysgenetic testis + ovotestis Pt 2: Testes	*NR5A1*: c.274C > T (p.Arg92Trp)
	[145]	Ambiguous	Pt 1: Testes Pt 2: Ovotestes, intratubular Pt 3: Histol. NA Pt 4: Ovotestis + ovary	*Pt 1 to 3*: *NR5A1* c.274C > T p.(Arg92Trp), *Pt 4:* *NR5A1* c.779C *>* T (p.Ala260Val).
	[147]	Ambiguous	Ovotestes	*NR5A1* c.275G > A (p.Arg92Gln)
*WT1* mutation	[148]	Ambiguous	Dysgenetic testes	*WT1*: c.1453_1456del; (p.Arg485Glyfs*14)
*NR2F2* mutation	[149]	Pt 1: Male genitalia, non-palpable gonads Pt 2 and Pt 3: Ambiguous genitalia	Pt 1 and Pt 2: Histol. NA Pt 3: Ovotestes	Pt 1: *NR2F2*: c.103_109delGGCGCCC (p.Gly35Argfs*75) Pt 2 and Pt 3: *NR2F2:* c.97_103delCCGCCCG (p.Pro33Alafs*77)

Pt: Patient Histol. NA: Histology not available.
